# Supplementary material for: Exploring the Genetic Relationship Between Type 2 Diabetes and Cardiovascular Disease: A Large-Scale Genetic Association and Polygenic Risk Score Study
Source: Biomolecules. 2024 Nov 18;14(11):1467. doi: 10.3390/biom14111467 (PMC11592259; doi:10.3390/biom14111467)
Supplement: Supplementary file 1 [file biomolecules-14-01467-s001.zip › biomolecules-3261129-supplementary.pdf]

Table S1. Disease identification using ICD-9, ICD-10 codes, and self-reported data.

**Disease**	**ICD-9**	**ICD-10**	**Self-reported**
Type-2 diabetes	25000,25010,25020,25090	E11	1223
Angina	413,4139	I20	1074
Atrial fibrillation	4273	I48	1471
Heart failure	428	I50	1076
Myocardial infarction	410-412	I21-25	1075
Peripheralvascular disease	440,443,444	I72-73	1067,1087,1088
Stroke	430,431,434,436	I60-64	1081,1086,1491,1583

Table S2. Genetic correlation (r_g) between type 2 diabetes (T2D) and various cardiovascular diseases (CVDs) subtypes with standard errors (SE) and p-values.

CVDs	$\mathbf{r_g}$	**SE**	*p*-values
Angina	0.379	0.053	1.31E-12
Atrial fibrillation	0.027	0.035	0.4399
Heart failure	0.422	0.152	0.0056
Myocardial infarction	0.327	0.038	1.12E-17
Peripheral vascular disease	0.321	0.115	0.0051
Stroke	0.427	0.078	3.67E-08

Table S3. Multivariable logistic regression analysis of atrial fibrillation (AF) risk across different PRS quartiles.

	PRS quartile	OR	95% CI		p-value
			Lower	Upper	
Model 1	PRS_quartileQ2	0.90	0.81	1.00	<0.05
	PRS_quartileQ3	0.84	0.75	0.93	<0.05
	PRS_quartileQ4	0.73	0.65	0.82	<0.001
Model 2	PRS_quartileQ2	0.91	0.82	1.01	9.01E-02
	PRS_quartileQ3	0.86	0.77	0.96	<0.05
	PRS_quartileQ4	0.75	0.67	0.84	<0.001
Model 3	PRS_quartileQ2	0.91	0.82	1.02	9.86E-02
	PRS_quartileQ3	0.86	0.77	0.96	<0.05
	PRS_quartileQ4	0.75	0.67	0.84	<0.001
Model 4	PRS_quartileQ2	0.92	0.83	1.02	1.19E-01
	PRS_quartileQ3	0.87	0.78	0.97	<0.05
	PRS_quartileQ4	0.76	0.68	0.85	<0.001
Model 5	PRS_quartileQ2	0.93	0.84	1.03	1.74E-01
	PRS_quartileQ3	0.88	0.79	0.98	<0.05
	PRS_quartileQ4	0.79	0.70	0.88	<0.001

Table S4. Multivariable logistic regression analysis of myocardial infarction (MI) risk across different PRS quartiles.

	PRS quartile	OR	95% CI		p-value
			Lower	Upper	
Model 1	PRS_quartileQ2	1.11	1.06	1.17	<0.001
	PRS_quartileQ3	1.22	1.16	1.28	<0.001
	PRS_quartileQ4	1.45	1.38	1.52	<0.001
Model 2	PRS_quartileQ2	1.13	1.08	1.19	<0.001
	PRS_quartileQ3	1.25	1.19	1.31	<0.001
	PRS_quartileQ4	1.46	1.39	1.53	<0.001
Model 3	PRS_quartileQ2	1.13	1.07	1.19	<0.001
	PRS_quartileQ3	1.24	1.18	1.31	<0.001
	PRS_quartileQ4	1.45	1.38	1.52	<0.001
Model 4	PRS_quartileQ2	1.11	1.06	1.17	<0.001
	PRS_quartileQ3	1.21	1.15	1.27	<0.001
	PRS_quartileQ4	1.37	1.30	1.43	<0.001
Model 5	PRS_quartileQ2	1.06	1.01	1.12	<0.05
	PRS_quartileQ3	1.10	1.05	1.16	<0.001
	PRS_quartileQ4	1.13	1.08	1.19	<0.001

Table S5. Multivariable logistic regression analysis of angina risk across different PRS quartiles.

	PRS quartile	OR	95% CI		p-value
			Lower	Upper	
Model 1	PRS_quartileQ2	1.11	1.05	1.17	<0.001
	PRS_quartileQ3	1.18	1.11	1.24	<0.001
	PRS_quartileQ4	1.44	1.36	1.52	<0.001
Model 2	PRS_quartileQ2	1.12	1.06	1.19	<0.001
	PRS_quartileQ3	1.20	1.13	1.27	<0.001
	PRS_quartileQ4	1.45	1.37	1.53	<0.001
Model 3	PRS_quartileQ2	1.12	1.06	1.19	<0.001
	PRS_quartileQ3	1.19	1.13	1.27	<0.001
	PRS_quartileQ4	1.44	1.36	1.52	<0.001
Model 4	PRS_quartileQ2	1.09	1.03	1.16	<0.001
	PRS_quartileQ3	1.14	1.07	1.21	<0.001
	PRS_quartileQ4	1.32	1.24	1.39	<0.001
Model 5	PRS_quartileQ2	1.04	0.98	1.10	2.18E-01
	PRS_quartileQ3	1.02	0.97	1.09	4.33E-01
	PRS_quartileQ4	1.05	0.99	1.11	8.31E-02

Table S6. Multivariable logistic regression analysis of heart failure (HF) risk across different PRS quartiles.

	PRS quartile	OR	95% CI		p-value
			Lower	Upper	
Model 1	PRS_quartileQ2	1.11	1.05	1.17	<0.001
	PRS_quartileQ3	1.18	1.11	1.24	<0.001
	PRS_quartileQ4	1.44	1.36	1.52	<0.001

Model 2	PRS_quartileQ2	1.12	1.06	1.19	<0.001
	PRS_quartileQ3	1.20	1.13	1.27	<0.001
	PRS_quartileQ4	1.45	1.37	1.53	<0.001
Model 3	PRS_quartileQ2	1.12	1.06	1.19	<0.001
	PRS_quartileQ3	1.19	1.13	1.27	<0.001
	PRS_quartileQ4	1.44	1.36	1.52	<0.001
Model 4	PRS_quartileQ2	1.09	1.03	1.16	<0.001
	PRS_quartileQ3	1.14	1.07	1.21	<0.001
	PRS_quartileQ4	1.32	1.24	1.39	<0.001
Model 5	PRS_quartileQ2	1.04	0.98	1.10	2.18E-01
	PRS_quartileQ3	1.02	0.97	1.09	4.33E-01
	PRS_quartileQ4	1.05	0.99	1.11	8.31E-02

Table S7. Multivariable logistic regression analysis of stroke risk across different PRS quartiles.

	PRS quartile	OR	95% CI		p-value
			Lower	Upper	
Model 1	PRS_quartileQ2	1.07	0.99	1.15	9.14E-02
	PRS_quartileQ3	1.13	1.05	1.21	<0.05
	PRS_quartileQ4	1.20	1.11	1.29	<0.001
Model 2	PRS_quartileQ2	1.07	0.99	1.16	6.85E-02
	PRS_quartileQ3	1.14	1.06	1.23	<0.001
	PRS_quartileQ4	1.20	1.11	1.29	<0.001
Model 3	PRS_quartileQ2	1.07	0.99	1.15	8.41E-02
	PRS_quartileQ3	1.13	1.05	1.22	<0.05
	PRS_quartileQ4	1.19	1.10	1.28	<0.001
Model 4	PRS_quartileQ2	1.06	0.98	1.14	1.54E-01
	PRS_quartileQ3	1.11	1.03	1.19	<0.05
	PRS_quartileQ4	1.15	1.07	1.24	<0.001
Model 5	PRS_quartileQ2	1.03	0.96	1.11	3.99E-01
	PRS_quartileQ3	1.05	0.98	1.13	1.83E-01
	PRS_quartileQ4	1.02	0.95	1.10	5.93E-01

Table S8. Multivariable logistic regression analysis of peripheral vascular disease (PVD) risk across different PRS quartiles.

	PRS quartile	OR	95% CI		p-value
			Lower	Upper	
Model 1	PRS_quartileQ2	1.11	0.91	1.36	3.08E-01
	PRS_quartileQ3	1.12	0.91	1.36	2.84E-01
	PRS_quartileQ4	0.86	0.69	1.06	1.60E-01
Model 2	PRS_quartileQ2	1.11	0.91	1.36	3.08E-01
	PRS_quartileQ3	1.12	0.91	1.36	2.83E-01
	PRS_quartileQ4	0.86	0.69	1.06	1.65E-01
Model 3	PRS_quartileQ2	1.11	0.91	1.35	3.18E-01
	PRS_quartileQ3	1.11	0.91	1.36	3.04E-01
	PRS_quartileQ4	0.86	0.69	1.06	1.54E-01

Model 4	PRS_quartileQ2	1.15	0.94	1.41	1.62E-01
	PRS_quartileQ3	1.19	0.97	1.45	9.28E-02
	PRS_quartileQ4	0.95	0.77	1.18	6.35E-01
Model 5	PRS_quartileQ2	1.15	0.94	1.41	1.63E-01
	PRS_quartileQ3	1.18	0.97	1.45	1.02E-01
	PRS_quartileQ4	0.94	0.75	1.16	5.55E-01

Table S9. Summary statistics of demographics and selected clinical measures.

	**T2D population**			**CVD population**		
	**Cases** *N* = **12,977**	**Controls** *N* = **352,031**		**Cases** *N* = **32,963**	**Controls** *N* = **332,045**	
**Demographic characteristics**						
Age			p<0.001			p<0.001
Mean (SD)	58.87 (7.23)	56.66 (8.03)		60.43 (6.79)	56.37 (8.04)	
Sex n (%)			p<0.001			p<0.001
Males	8232 (63.44%)	160,378 (43.94%)		22,135 (67.15%)	146,475 (44.11%)	
Females	4745 (36.56%)	191,653 (54.44%)		10,828 (32.5%)	185,570 (55.89%)	
Level of education n (%)			p<0.001			p<0.001
7yrs	3241 (24.97%)	58,491 (16.62%)		9053 (27.46%)	52,679 (15.87%)	
10yrs	3498 (26.96%)	96,046 (27.28%)		7857 (23.84%)	91,687 (27.61%)	
13yrs	1471 (11.34%)	43,465 (12.35%)		3521 (10.68%)	41,415 (12.47%)	
15yrs	760 (5.86%)	17,809 (5.06%)		1884 (5.72%)	16,685 (5.02%)	
19yrs	1173 (9.04%)	22,852 (6.49%)		2778 (8.43%)	21,247 (6.40%)	
20yrs	2834 (21.84%)	113,368 (32.20%)		7870 (23.88%)	108,332 (32.63%)	
**Hospital admission examinations**						
Body mass index (BMI)			p<0.001			p<0.001
Mean (SD)	31.31 (5.43)	27.26 (4.67)		28.62 (4.86)	27.28 (4.73)	
Systolic blood pressure (SBP)	144.47 (18.24)	139.70 (19.02)	p<0.001	141.65 (19.03)	139.69 (19.00)	p<0.001
Diastolic blood pressure (DBP)	83.95 (10.27)	82.17 (10.31)	p<0.001	81.33 (10.59)	82.32 (10.29)	p<0.001
**Lifestyle factors**						
Smoking n (%)			p<0.001			p<0.001
Yes	1588 (12.24%)	35,646 (10.13%)		4175 (12.67%)	33,059 (9.96%)	

No	11389 (87.76%)	316,385 (89.87%)		28,788 (87.33%)	298,986 (90.04%)	
Drinking n (%)			p<0.001			p<0.001
Yes	12,265 (94.51%)	339,748 (96.51%)		31,597 (95.86%)	320,416 (96.50%)	
No	712 (5.49%)	12,283 (3.49%)		1366 (4.14%)	11,629 (3.50%)	
**Biological variables**						
Red blood cell count (RBC)	4.65 (0.40)	4.51 (0.40)	p<0.001	4.57 (0.41)	4.51 (0.40)	p<0.001
White blood cell count (WBC)	7.54 (1.85)	6.85 (2.00)	p<0.001	7.24 (1.97)	6.84 (2.00)	p<0.001
Platelet count (PLT)	247.33 (59.78)	253.26 (58.95)	p<0.001	243.34 (59.73)	254.01 (58.83)	p<0.001
Creatinine	74.24 (18.76)	72.28 (17.58)	p<0.001	78.16 (22.48)	71.77 (16.97)	p<0.001
Cholesterol	5.11 (1.23)	5.72 (1.11)	p<0.001	5.13 (1.22)	5.76 (1.09)	p<0.001
High-density lipoprotein (HDL) cholesterol	1.23 (0.29)	1.45 (0.36)	p<0.001	1.31 (0.33)	1.46 (0.36)	p<0.001
Low-density lipoprotein (LDL) cholesterol	3.17 (0.92)	3.58 (0.84)	p<0.001	3.17 (0.92)	3.60 (0.83)	p<0.001
Triglycerides (TG)	2.27 (1.27)	1.72 (0.98)	p<0.001	1.89 (1.07)	1.72 (0.99)	p<0.001
C-reactive protein (CRP)	3.40 (4.65)	2.50 (4.26)	p<0.001	2.88 (4.90)	2.49 (4.21)	p<0.001
Glycated haemoglobin (HbA1c)	46.30 (12.37)	35.63 (5.84)	p<0.001	38.34 (9.05)	35.78 (6.14)	p<0.001
Apolipoprotein A (Apo-A)	0.97 (0.25)	1.04 (0.23)	p<0.001	0.95 (0.24)	1.04 (0.23)	p<0.001
Apolipoprotein B (Apo-B)	1.52 (0.28)	1.54 (0.27)	p<0.001	1.51 (0.28)	1.54 (0.27)	p<0.001
Vitamin D	44.22 (19.10)	49.29 (20.11)	p<0.001	5.13 (1.22)	5.76 (1.09)	p<0.001

* Continuous variables are summarised by mean (standard deviation) and compared using one-way ANOVA. Categorical variables are summarised by number (percentage) and compared using Pearson's $\chi 2$ test.
